# Peri- and postmenopause—diagnosis and interventions interdisciplinary S3 guideline of the association of the scientific medical societies in Germany (AWMF 015/062): short version

**DOI:** 10.1007/s00404-020-05682-4

**Published:** 2020-07-13

**Authors:** Olaf Ortmann, Maria J. Beckermann, Elisabeth C. Inwald, Thomas Strowitzki, Eberhard Windler, Clemens Tempfer, For the guideline group

**Affiliations:** 1 Department of Gynecology and Obstetrics, University Medical Center Regensburg, Landshuter Straße 65, 93053 Regensburg, Germany; 2 Frauenärztliche Gemeinschaftspraxis, Köln, Germany; 3 Department of Gynecological Endocrinology and Fertility Disorders, University Women’s Hospital, Heidelberg, Germany; 4 Endocrinology and Metabolism of Ageing, University Medical Center Hamburg-Eppendorf, Hamburg-Eppendorf, Germany; 5 Department of Obstetrics and Gynecology, Ruhr-University Bochum, Bochum, Germany

**Keywords:** Perimenopause, Postmenopause, Hormone replacement therapy, Diagnosis, Treatment, Risk communication

## Abstract

This short version of the interdisciplinary S3 guideline “Peri- and Postmenopause—Diagnosis and Interventions” is intended as a decision-making instrument for physicians who counsel peri- and postmenopausal women. It is designed to assist daily practice. The present short version summarizes the full version of the guideline which contains detailed information on guideline methodology, particularly regarding the critical appraisal of the evidence and the assignment of evidence levels. The statements and recommendations of the full version of the guideline are quoted completely in the present short version including levels of evidence (LoE) and grades of recommendation. The classification system developed by the Centre for Evidence-based Medicine in Oxford was used in this guideline.

**Electronic supplementary material:**

The online version of this article (10.1007/s00404-020-05682-4) contains supplementary material, which is available to authorized users.

## Introduction

Peri- and postmenopausal women often seek medical assistance because of climacteric symptoms (e.g. vasomotoric symptoms). They want information (a) about the physiological changes during the menopausal transition period and (b) ways to alleviate their symptoms to improve their quality of life. During peri- and postmenopause, symptoms may change. Dysfunctions and diseases may develop that may or may not depend on the changes of sex hormones. Perimenopausal women often ask for information about how to prevent diseases that are typically associated with the perimenopausal transition and the postmenopause. The current S3 guideline, Peri- and Postmenopause—Diagnosis and Interventions’ is based on the previous interdisciplinary S3 guideline, Hormone therapy in perimenopause and postmenopause’ [1, Appendix]. The authors of the current S3 guideline intended to take a broader spectrum of issues of the peri- and postmenopause into account and provide evidence-based recommendations to counsel women in this period of life.

## Diagnosis and treatment

**Table:** 

Evidence-based recommendation		
Level of Evidence LLA	Grade of recommendation A	Degree of consensus++
Peri- and Postmenopause in women aged 45 or older should be diagnosed based on clinical parameters

**Table:** 

Evidence-based recommendation		
Level of Evidence LLA	Grade of recommendation A	Degree of consensus++
FSH should be used to diagnose peri- and postmenopause only in women between 40 and 45 years with climacteric symptoms or in women under the age of 40 years with suspected indicators of premature ovarian insufficiency		

The different phases of the menopausal transition can be mainly diagnosed by clinical criteria. Hormone measurements are usually not necessary [1, 2]. The following terms should be used for healthy women older than 45 years who have climacteric symptoms without laboratory tests:

- Perimenopause—women with irregular menstrual cycles and possibly vasomotor symptoms.
- Postmenopause—women without a menstrual bleeding for at least 1 year in the absence of interventions which may cause amenorrhea (e.g. oral contraceptives).
- Peri-/postmenopause—women after hysterectomy with vasomotor symptoms. In women who are using hormone replacement therapy (HRT), it may be difficult to define the menopausal status.

In women older than 45 years, measurements of anti-Müllerian hormone, inhibin A, inhibin B, antral follicle count or ovarian volume should not be used to diagnose peri- or postmenopause. Follicle stimulating hormone (FSH) measurement may be used in women between 40 and 45 years with climacteric symptoms or irregular menstrual cycles and in women younger than 40 years with suspected premature ovarian insufficiency [1, 2].

### Symptoms

Vasomotor symptoms such as hot flushes and night sweats are the most common symptoms of the climacteric syndrome in peri- and postmenopausal women. Other symptoms such as sleep disturbances, mood swings, anxiety, and sexual impairment may also be associated with the peri- and postmenopause. In contrast to the vasomotoric symptoms, it is not clear whether the other symptoms are caused by hormonal changes. Loss of estradiol is the main cause of the climacteric syndrome, but the exact role of serum and tissue level changes of estradiol and other hormones such as androgens and gonadotropins is unclear.

Frequency and duration of all climacteric symptoms vary considerably and may be influenced by a number of factors such as general health and well-being, socioeconomic status, cultural influence, and other factors. The prevalence of hot flushes has been reported to be between 14 and 51% among premenopausal women, about 50% among perimenopausal women, and between 30 and 80% among postmenopausal women [3]. The median duration of hot flushes is 7.4 years. Longer durations typically occur when climacteric symptoms already develop in the premenopause [4].

### Information

Peri- and postmenopausal women should be informed about the phases of peri- and postmenopause, the diagnosis, typical symptoms (vasomotor symptoms, sleep disorders, mood changes, urogenital symptoms, sexual impairment, changes in lifestyle and other measures which may affect health and well-being). In addition, they should be informed about the benefits and risks of different treatments strategies and the long-term effects of peri- and postmenopause on female health. Peri- and postmenopausal women should also be informed about different treatment options such as hormonal interventions, non-hormonal interventions and non-pharmacologic interventions.

## Therapeutical interventions

**Table:** 

Evidence-based recommendation		
Level of Evidence 1a	Grade of recommendation A	Degree of consensus + +
Women with vasomotor symptoms should be offered hormone replacement therapy (HRT) after information about short- (up to 5 years) and long-term risks and benefits. Women with an intact uterus may receive estradiol replacement therapy with an appropriate progestin component (EPT), women after hysterectomy may receive estradiol replacement therapy (ET) only

**Table:** 

Evidence-based recommendation		
Level of Evidence 3	Grade of recommendation A	Degree of consensus + +
Serotonin reuptake inhibitors (SSRIs), serotonin noradrenaline reuptake inhibitors (SNRIs), clonidine, and gabapentin should not be offered as treatment of first choice for the treatment of vasomotor symptoms

**Table:** 

Evidence-based recommendation		
Level of Evidence 1b	Grade of recommendation 0	Degree of consensus + +
Cognitive behavioural therapy (CBT), isoflavones, and cimicifuga preparations may be used to treat vasomotor symptoms		
* See “Appendix” for dissenting opinion of the GPT		

## Vasomotoric symptoms

Estradiol is the most efficient means of therapy of climacteric symptoms. Therefore, HRT should be offered all women with moderate to severe climacteric symptoms with significant impairment of their quality of life. For women with an intact uterus, EPT is the most efficient treatment of vasomotoric symptoms. Hysterectomized women should only receive ET. The progestin component of EPT can be applied cyclically or continuously. Potent progestins such as synthetic progestins should be used for at least 10–12 days per month. Shorter treatment periods, or the use of natural progesterone and dydrogesterone instead of synthetic progestins may increase the risk of endometrial hyperplasia and endometrial cancer. One must consider that different HRT preparations or routes of application have different risk profiles. Transdermal application may have a better risk–benefit ratio and should be preferred over oral preparations. Women who desire phytotherapy should be informed that numerous preparations exist, and that the safety of these preparations is often unclear. In addition, combinations of different phytotherapy compounds may have different effects and may interact with concurrent medications [1, 2].

## Changes of sexual function

**Table:** 

Evidence-based recommendation		
Level of Evidence 1b	Grade of recommendation 0	Degree of consensus + +
Peri- and postmenopausal women with loss of libido can be offered testosterone treatment after psychosexual exploration and when HRT has not been efficient. They should be informed about off-label use		

Peri- and postmenopausal women may observe changes in sexual function. They should be asked about signs of vaginal atrophy such as dyspareunia. Loss of libido is common in ageing women. Low levels of estrogen and testosterone may be associated with loss of libido. However, there is no strict association between sex hormone levels and libido as reasons for loss of libido are complex. A psychotherapeutic intervention may be beneficial. Treatment with testosterone may be considered when HRT was not efficient for improving libido. Moisturizers and lubricants may be used alone or in addition to vaginal estrogen preparations. All these interventions are usually sufficient to reduce vaginal symptoms such as dryness, pain on intercourse or vaginal discharge. When estrogens are used for the treatment of vaginal dryness, estriol (E3)-containing preparations should be preferred. E3 may be used efficiently in low or ultra-low doses. The dosage may be increased if symptoms are not relieved. When treatment is terminated, symptoms typically re-occur. Side effects of vaginal ET are rare [1, 2].

## Urogenital atrophy

**Table:** 

Evidence-based recommendation		
Level of Evidence 1b	Grade of recommendation A	Degree of consensus +++
Women with symptomatic urogenital atrophy should be offered lubricants alone or together with a vaginal ET. The treatment duration should be individualized		

## Starting and stopping HRT

After starting HRT, women should consult their gynaecologist regularly (initially after 3 months) to monitor treatment effectiveness and tolerability of the therapy. Women should be advised to consult their gynaecologist in case of atypical bleeding. Modification of HRT has to be considered for several reasons during different phases of peri- and postmenopause. When treatment is well tolerated and no pathological symptoms occur, yearly gynecologic consultations are appropriate. Vaginal ultrasound examinations and endometrial thickness measurements should not be used routinely during HRT [5].

If it is determined that HRT should be stopped, two options can be offered: gradually reducing HRT or ceasing immediately. Gradually reducing may limit recurrence of symptoms in the short term. Whichever method is chosen, it has no influence on the recurrence rate of symptoms in the longer term.

## Urogynecology

### Urinary incontinence

**Table:** 

Evidence-based statement		
Level of Evidence 1a		Degree of consensus + +
Vaginal ET may improve urinary incontinence in postmenopausal women

**Table:** 

Evidence-based recommendation		
Level of Evidence 1a	Grade of recommendation A	Degree of consensus + +
Patients should be informed before systemic ET or EPT that these therapies may lead to occurrence or worsening of urinary incontinence

**Table:** 

Evidence-based recommendation		
Level of Evidence 1a	Grade of recommendation A	Degree of consensus + +
Postmenopausal women with urinary incontinence should be offered pelvic floor training and vaginal ET		

Randomised controlled trials have shown that in women with urinary incontinence, vaginal ET leads to significant improvements compared to placebo. However, a significantly higher prevalence of incontinence was documented when ET was applied systemically. Specifically, prevalence of urinary incontinence was doubled compared to placebo. EPT also leads to increases of urinary incontinence. However, the effect was smaller than in ET users [6, 11].

### Overactive bladder

**Table:** 

Evidence-based statement		
Level of Evidence 1b		Degree of consensus +++
Systemic HRT may lead to worsening of urinary incontinence. Women with overactive bladder may be offered vaginal ET

**Table:** 

Evidence-based recommendation		
Level of Evidence 1b	Grade of recommendation 0	Degree of consensus + +
After exclusion of urological diseases, women with symptoms of urgency and frequency may be offered local ET		

### Urinary tract infections

**Table:** 

Evidence-based statement		
Level of Evidence 2b		Degree of consensus + +
Changes in the vaginal milieu of postmenopausal women may predispose to urinary tract infection, especially in older women

**Table:** 

Evidence-based recommendation		
Level of Evidence 2a	Grade of recommendation B	Degree of consensus + +
Recurrent urinary tract infections in postmenopausal women should preferably be treated with vaginal ET instead of antibiotics		

Two small studies have demonstrated that vaginal ET reduced the frequency of urinary tract infections. Oral ET did not lead to a reduction of recurrent urinary tract infections [6, 8, 12, 16].

## Cardiovascular disease

**Table:** 

Evidence-based recommendation		
Level of Evidence 2b	Grade of recommendation B	Degree of consensus + +
The risk for cardiovascular disease in peri- and postmenopausal women varies depending on their risk profile. These risk factors should be controlled optimally to exclude contraindications for HRT. Therefore, cardiovascular risk factors have to be identified and treated before HRT is initiated		

Because of the various limitations of the trials, mainly the WHI, statements and recommendations on the effects of HRT on cardiovascular diseases are based on a moderate level of evidence. Large randomized intervention trials have shown no general protection against cardiovascular diseases by HRT but indicate a neutral or even negative effect of HRT in postmenopausal women [17]. The analyses of data including recent studies indicate the need for caution, but individualized treatment may have little risk or even cardiovascular benefit. The transdermal application has advantages over the oral form [18]. In any case, the prerequisite seems to be the control of the conventional risk factors for cardiovascular events such as heart attack and stroke. Regardless of the study results, previous vascular events may be contraindications for HRT [1, 2].

## Thromboembolism

**Table:** 

Evidence-based recommendation		
Level of Evidence 2a	Grade of recommendation A	Degree of consensus + +
Women should be informed that risk of thromboembolism is higher during oral compared to transdermal ET and EPT		

The highest vascular risks of oral HRT are venous thrombosis and thromboembolism [1, 2, 19, 21]. ET and EPT double the risk by about two cases per 1000 women per year [22, 25]. Estrogens and progestogens have thrombogenic effects that are dose dependent and substance specific, which are more pronounced in the initial phase of therapy and increase with age, weight and genetic predisposition [20, 21]. With low-dose transdermal therapy, there was no evidence of an increased risk of thromboembolism probably due to the lack of a first-pass effect in the liver [19, 21].

## Cerebrovascular events

**Table:** 

Evidence-based recommendation		
Level of Evidence 2b	Grade of recommendation A	Degree of consensus + +
Women should be informed that oral EPT, but not transdermal ET may increase the risk for ischemic cerebrovascular events. Absolute risk for cerebral stroke is very low in younger women		

Ischemic strokes are the second most common and because of possible persistent disabilities very serious risk of oral HRT [2, 18, 19, 26]. In absolute numbers, the risk increases by 1 case per 1000 women per year during oral ET or EPT intake [22, 27, 28]. An increase in the risk of stroke may be avoidable using transdermal HRT up to a dose of 50 µg [1, 2].

## Coronary heart disease

**Table:** 

Evidence-based recommendation		
Level of Evidence 2b	Grade of recommendation A	Degree of consensus + +
Women should be informed that EPT does not increase their cardiovascular risk or has only a minor effect. ET does not increase cardiovascular risk or may even reduce it. HRT is not appropriate for the primary or secondary prevention of coronary heart disease. HRT should be started before the age of 60 years for the treatment of climacteric symptoms		

Neither ET nor EPT have significant influences on the risks for coronary heart disease in primary and secondary prevention [17, 22, 23, 25]. However, HRT initiated within the first 10 years after menopause appears to be associated with a lower coronary event rate, while starting 20 years or more after menopause significantly increases the risk [1, 2, 19, 25].

## Osteoporosis

**Table:** 

Evidence-based statement		
Level of Evidence 1a		Degree of consensus +++
HRT significantly reduces the risk for osteoporotic fractures

**Table:** 

Evidence-based statement		
Level of Evidence 2a		Degree of consensus + +
The reduction of osteoporotic fracture risk by HRT is independent of treatment duration (even treatment duration of less than 1 year leads to reduction of the risk of osteoporotic fractures) and age at initiation of HRT. In addition, risk reduction seems to persist after stopping HRT		

## Prevention

Excessive weight reduction leads to an increased risk for atraumatic fractures. A body mass index below 20 should be avoided. Consequently, weight gain is associated with reduced risk of atraumatic fractures. Weight gain above a body mass index of 35 should be avoided. Exercise and sufficient intake of calcium and vitamin D is recommended [1, 2, 29, 32].

## Treatment

A number of therapies are licensed for the treatment of osteoporosis: estrogen (in combination with progestin in non-hysterectomised women), bisphosphonates, selective estrogen receptor modulators (SERMs), and a human monoclonal antibody against the RANK ligand (Denosumab) and parathyroid hormone (teriparatid). The efficiency of EPT or ET for primary prevention of osteoporotic factors has been demonstrated in observational studies and randomized controlled trials. The current version of the S3 Guideline of the German Association of Osteology (DVO) recommends that prevention or treatment of osteoporosis by HRT is feasible when women have concurrent climacteric symptoms or do not tolerate or have contraindications against licensed treatments for the prevention or treatment of osteoporosis [1, 2, 29, 32].

The reduction of fractures by HRT is independent of the duration of HRT and the age at treatment initiation. In addition, the risk reducing effect of HRT seems to persist at a lower level after its termination. Since osteoporosis is a chronic disease, treatment is usually performed for longer durations depending on the assumed baseline risk for fractures. The mean duration is 3–4 years. In accordance to the results of the WHI, the following risk reducing effects can be expected: − 23 fractures per 1000 HRT users (confidence interval [CI] 10–33), − 20 non-vertebral fractures per 1000 HRT users (CI 6–30), − 8 vertebral fractures per 1000 HRT users (CI − 18 to + 9), − 0 hip fractures per 1000 HRT users (CI − 10 to + 4), − 36 wrist fractures per 1000 HRT users (CI 19–43) [1, 2]. In women with premature ovarian insufficiency, HRT or oral contraceptives should be used until the age of natural menopause to prevent osteoporosis and osteoporotic fractures.

## Dementia, depression and mood changes

**Table:** 

Evidence-based recommendation		
Level of Evidence LLA	Grade of recommendation A	Degree of consensus +++
Peri- and postmenopausal women should be informed that it is unclear whether HRT before the age of 65 has an influence on the risk of dementia.Peri- and postmenopausal women should be informed that it is unclear whether HRT before the age of 65 has an influence on the risk of dementia		

**Table:** 

Evidence-based recommendation		
Level of Evidence LLA	Grade of recommendation A	Degree of consensus + +
Pharmacological treatment of depression in perimenopausal women should be used according to current treatment guidelines. Currently, there is no evidence indicating that the efficacy of anti-depressant medications varies according to menopausal status. There is insufficient evidence to recommend HRT or psychotherapy for the treatment of perimenopausal depression		

There are no data with strong evidence for an increase for the risk or a beneficial effect on dementia in women using HRT before the age of 65 years [2]. In the WHI, it was demonstrated that HRT when initiated after the age of 65 years increased the risk for dementia significantly [22]. HRT may be considered for mood changes which develop or worsen during the menopausal transition. There is no evidence for an effect of selective serotonin reuptake inhibitors (SSRI) or selective noradrenalin reuptake inhibitors (SNRI) on menopausal mood changes [2].

There is no convincing data for the efficacy of HRT in prevention or treatment for depression in peri- und postmenopausal women. Women with depression should be treated according to current treatment guidelines [33, 34].

## HRT and cancer risk

### HRT and breast cancer risk

**Table:** 

Evidence-based recommendation		
Level of Evidence 1a	Grade of recommendation A	Degree of consensus + +
Women considering HRT should be informed that HRT (EPT/ET) may lead to a small or no increased risk of breast cancer. Breast cancer risk depends on HRT formulations and treatment duration and is reduced after the end of treatment		

### EPT

EPT may increase breast cancer risk. This was shown in a number of meta-analyses that included observational studies as well as randomized controlled trials (RCTs). In the randomized Women´s Health Initiative (WHI) trial, EPT led to an increased relative risk of breast cancer of 1.26 with 8 additional cases per 10,000 women per year [22, 35]. Cohort studies have shown that continuous combined EPT leads to higher increases in breast cancer risk than sequential EPT. RCTs and observational studies demonstrated that current users of EPT have an increased breast cancer risk.

However, this risk is reduced after stopping HRT. When HRT was initiated at or around the age of menopause, breast cancer risk increases were higher than in women who initiated HRT more than 5 years after menopause. EPT-containing progesterone leads to smaller increases of breast cancer risk compared to EPT-containing synthetic progestins [36]. However, progesterone has a smaller anti-proliferative effect on the endometrium than synthetic progestins. In the WHI, study breast cancer mortality was increased in HRT users in the short term, but not after 18 years of follow-up [1, 2, 37].

### ET

In contrast to a large number of observational studies, the WHI trial demonstrated a significant risk reduction for breast cancer in ET versus placebo users [38]. The mean duration of ET was 7 years. Three smaller RCTs did not find significant differences in breast cancer risk of women using ET compared to placebo [39, 41]. Observational studies have shown a small increase in breast cancer risk in women using ET. The duration of ET treatment leading to an elevated breast cancer risks is controversial. Breast cancer-specific mortality was reduced in ET uses in the 18-years follow-up in the WHI trial [37]. However, this calculation is based on a small number of breast cancer cases.

In summary, HRT may be associated with increased breast cancer risk. The elevation is relatively small and has to be included in the individual risk–benefit calculation before initiating HRT for climacteric symptoms.

## Vaginal ET and breast cancer risk

Vaginal ET may lead to increases of systematic estrogen levels. It is not known whether it may also lead to increases of breast cancer risk. Ultra-low doses of vaginal ET (e.g. 0.03 mg estriol, 2–3 applications per week) lead to good clinical effects on vaginal symptoms. It is unlikely that such an ultra-low-dose vaginal ET has a causal influence on breast cancer risk even when the therapy is used chronically [42].

## HRT after breast cancer

**Table:** 

Evidence-based statement		
Level of Evidence 2b		Degree of consensus +++
HRT may increase the risk of relapse after breast cancer

**Table:** 

Evidence-based recommendation		
Level of Evidence 2a	Grade of recommendation A	Degree of consensus +++
HRT should not be used in women after breast cancer. In selected cases it may be used after ineffective non-hormonal treatments and severe limitations of quality of life		

Meta-analyses including observational studies and RCTs could not demonstrate an increased risk of relapse of breast cancer after using HRT [43, 46]. However, these studies have considerable methodological limitations such as low numbers of cases and short follow-up. The HABITS trial, an RCT, demonstrated an increase of breast cancer relapse in breast cancer survivors using HRT [44, 45]. However, the number of only 442 women in this study was small. The Stockholm randomized trial with 378 breast cancer survivors found no increased risk of relapse in the HRT arm. Therefore, it is not possible to make reliable conclusions on the oncological safety of HRT among breast cancer survivors. In the randomised controlled LIBERATE trial 3000 women with vasomotoric symptoms after breast cancer were treated with tibolone or placebo. Tibolone led to increased risk of breast cancer (hazard ratio [HR] 1.40) after a median follow-up of 3.1 years [47].

## Vaginal ET after breast cancer

Women who have been or are being treated for breast cancer may complain about vaginal symptoms such as dryness, dyspareunia, and pain. They use vaginal ET six times more often than systemic HRT. Vaginal ET may lead to increases in serum estrogen levels. Vaginal ETs are different in type, dosage, and frequency of application. In Germany, estriol is frequently used for vaginal ET. Ultra-low-dose estriol in combination with lactobacillus acidophilus was tested in a small study on its safety and efficacy. In this study, vaginal atrophy was significantly improved and serum levels of estriol were only increased during the initial period of ET. After 4 weeks of treatment, levels of estriol were slightly elevated in 50% of users while not at all in the remaining 50%. It can be concluded that ultra-low-dose vaginal estriol therapy (0.03 mg, 3 applications per week) may be used in breast cancer survivors if non-hormonal alternatives had insufficient results [48].

## HRT and endometrial cancer

**Table:** 

Evidence-based statement		
Level of Evidence 2		Degree of consensus +++
HRT containing an estrogen without a progestin is a risk factor for endometrial cancer in non-hysterectomized women. The effect is dependent on the duration of treatment

**Table:** 

Evidence-based statement		
Level of Evidence 2		Degree of consensus + +
Continuous combined HRT with conjugated equine estrogens and medroxyprogesterone acetate for an average duration of 5.6 years reduced the risk of endometrial cancer

**Table:** 

Evidence-based statement		
Level of Evidence 2		Degree of consensus + +
Continuous combined HRT for less than 5 years does not increase risk of endometrial cancer

**Table:** 

Evidence-based statement		
Level of Evidence 3		Degree of consensus + +
Long-term treatment with of continuous combined HRT for more than 6 or 10 years may lead to an increased risk of endometrial cancer

**Table:** 

Evidence-based statement		
Level of Evidence 4		Degree of consensus +
The use of progesterone or dydrogesterone in continuous combined HRT may increase the risk of endometrial cancer

**Table:** 

Evidence-based statement		
Level of Evidence 3		Degree of consensus +++
Sequential combined HRT may increase the risk of endometrial cancer. The effect depends on duration, type and dose of progestin

**Table:** 

Evidence-based statement		
Level of Evidence 3	Grade of recommendation A	Degree of consensus +++
Sequential combined HRT for a duration of less than 5 years including a synthetic progestin does not increase the risk of endometrial cancer risk

**Table:** 

Evidence-based recommendation		
Level of Evidence LLA	Grade of recommendation A	Degree of consensus + +
ET should only be performed in hysterectomized women. A combined EPT in non-hysterectomized women should contain a progestin for a duration of 10, better 14 days per treatment month		

ET may lead to an increased risk of endometrial cancer in postmenopausal, non-hysterectomized women. This effect is dependent on duration and dose of ET [5]. Standard doses (e.g. 2 mg estradiol, 0.625 mg conjugated equine estrogen) increase the relative risk of endometrial cancer after more than 3 years of use up to five times and after 10 years of use up to ten times. This effect persists for several years after stopping ET. Therefore, HRT should include at least 1 mg norethisterone or 2 mg medroxyprogesterone acetate or an equivalent progestin in combined continuous HRT users or at least 5 mg medroxyprogesterone acetate or an equivalent progestin in sequential HRT users with a sufficient duration of progestin treatment (10–15 days per treatment month) [49, 52]. EPT is performed in a sequential or continuous combined form. Sequential EPT containing at least 10 days of progestin treatment (for up to 5 years) did not increase the risk of endometrial cancer [53]. Longer sequential EPT may increase endometrial cancer risk. The reduction of progestin use for less than 10 days per month significantly increases endometrial cancer risk (RR 3.0–4.4) [51, 54]. The progestin component of EPT was shown to influence the risk of endometrial cancer in the prospective E3N cohort study. In this study, long-term application of progesterone or dydrogesterone increased endometrial cancer risk (progesterone HR 2.66 after more than 5 years of treatment; dydrogesterone HR 1.69 after more than 5 years of treatment) [55]. Continuous combined EPT with conjugated equine estrogen and medroxyprogesterone acetate led to reduced risk for endometrial cancer [56]. In observational studies such as the Million Women Study and the EPIC study, a significantly reduced endometrial cancer risk was found in continuous combined HRT users [52, 53]. Other studies, however, documented significant risk increases [51, 54].

## Vaginal ET and endometrial cancer risk

Clinical studies did not show increased rates of endometrial hyperplasia after vaginal ET. Therefore, it is not recommended to add a progestin to vaginal ET. However, there are no data on endometrial safety of vaginal ET when used for more than 1 year [5].

## HRT after endometrial cancer

**Table:** 

Evidence-based statement		
Level of Evidence 2b		Degree of consensus +++
The effect of HRT on the risk of relapse after treatment of endometrial cancer has not been investigated sufficiently

**Table:** 

Evidence-based recommendation		
Level of Evidence 2b	Grade of recommendation EK	Degree of consensus + +
In patients who have been treated for endometrial cancer, HRT may be used for the treatment of climacteric symptoms if they have severe quality of life limitations and in whom non-hormonal treatments were not effective		

A recent meta-analysis has included only one randomized trial and five observational studies assessing the safety of HRT in women who have been treated for endometrial cancer. Of note, the risk of endometrial cancer relapse was reduced in HRT users. However, most studies included in this meta-analysis were retrospective observational studies. Therefore, the evidence regarding the oncological safety of HRT among endometrial cancer survivors is limited. It can only be concluded that HRT after early stage endometrial cancer may not lead to a relevant increase of the risk of relapse [5, 57, 59].

## Vaginal ET after endometrial cancer

**Table:** 

Evidence-based recommendation		
Level of Evidence 4	Grade of recommendation A	Degree of consensus + +
Patients with symptoms of vaginal atrophy after treatment of endometrial cancer should be treated primarily with moisturizers or lubricants

**Table:** 

Consensus-based recommendation		
EK		Degree of consensus + +
After treatment of endometrial cancer, vaginal ET may be used if lubricants or cremes have proven to be ineffective		

Patients who have been treated for endometrial cancer may complain vaginal dryness, pain, dyspareunia, vaginal bleeding, and urinary incontinence. This may lead to sexual dysfunction and reduction of quality of life. Since it cannot be excluded that vaginal ET may lead to increase the risk of relapse, endometrial cancer survivors should be treated with non-hormonal moisturizers or lubricants [60]. Ph-stabilizing preparations with a pH between 4 and 4.5 have been shown to be efficient in this indication [61]. Local ET may alleviate the symptoms of vaginal atrophy after radiotherapy of the vagina [62]. Retrospective observational studies did not show increased rates of relapse after vaginal ET [63]. However, the evidence is limited and does not prove oncological safety [5].

## HRT and ovarian cancer risk

**Table:** 

Evidence-based recommendation		
Level of Evidence 2a	Grade of recommendation A	Degree of consensus + +
Women considering HRT should be informed that ET and EPT may increase ovarian cancer risk. This effect has been observed after treatment durations of less than 5 years and is reduced after stopping treatment		

The Collaborative Group on Epidemiological Studies of Ovarian Cancer performed a meta-analysis of 52 studies. This included data from 21 488 postmenopausal women with ovarian cancer. Data from prospective trials showed that HRT users had increased risk for ovarian cancer after HRT durations of less than 5 years (RR 1.43). The absolute increase of risk is 1 in 1000 after 5 years and 1 in 1600 women using HRT [64].

## HRT after ovarian cancer

**Table:** 

Evidence-based statement		
Level of Evidence 2b		Degree of consensus + +
The safety of HRT after treatment of ovarian cancer is unclear

**Table:** 

Evidence-based recommendation		
Level of Evidence 2b	Grade of recommendation 0	Degree of consensus +++
HRT may be used after treatment of ovarian cancer and appropriate consultation with the patient		

After treatment, ovarian cancer patients may experience natural or therapy-induced menopause. These patients may suffer from climacteric symptoms. Younger women may develop estrogen-associated diseases such as coronary heart disease and osteoporosis [65, 70]. There are only few studies that examined the safety of HRT after ovarian cancer [71]. Three observational studies could not demonstrate increased risk of relapse [66, 68, 69]. However, there are a number of methodological limitations. A randomized study with 150 patients showed improved survival in the group of women treated with HRT after a median follow-up of 19.1 years (HR 0.63; 95% CI 0.44–0.90). The authors conclude that HRT after ovarian cancer is safe. However, this study has a number of limitations and the safety of HRT after ovarian cancer remains unclear [67]. Since none of the studies showed an increased, but rather a risk reduction, HRT may be considered when women complain of severe climacteric symptoms. Especially in younger women with iatrogenic menopause after ovarian cancer treatment, the risk of mortality due to coronary heart disease is increased. In these women ET should be considered.

## HRT and risk for colorectal cancer

**Table:** 

Evidence-based recommendation		
Level of Evidence 2a	Grade of recommendation A	Degree of consensus +++
Women should be informed that HRT may reduce the risk of colorectal cancer. HRT should not be used for colorectal cancer prevention		

Reproductive factors may influence the risk of colorectal cancer. Studies have shown that HRT has no effect or reduces the risk for colorectal cancer [72, 73]. In the WHI trial, the risk of colon cancer was reduced by 37% in EPT users. ET did not have any effect on colorectal cancer risk [74]. Observational studies had conflicting results. The majority of these studies showed a risk reduction after HRT use. A meta-analysis including data from RCTs and observational studies showed that EPT as well as ET significantly reduced the risk of colorectal cancer (RR 0.74; 95% CI 0.68–0.81 and RR 0.79; 95% CI 0.69–0.91, respectively). A recent publication of data from five Danish cancer registries including 1.1 million women also demonstrated that ET and EPT reduced the risk of colorectal cancer with a minimal effect of EPT on rectal cancer [73].

## Premature ovarian insufficiency (POI)

**Table:** 

Evidence-based recommendation		
Level of Evidence 2b	Grade of recommendation B	Degree of consensus + +
Women with POI should be informed about the importance of HRT or combined oral contraceptives at least until the age of natural menopause if there are no contraindications against HRT or combined oral contraceptives

**Table:** 

Evidence-based statement		
Level of Evidence 2b		Degree of consensus + +
There is no evidence for different treatment efficacy of HRT and combined oral contraceptives in women with POI		

Premature ovarian insufficiency (POI) or premature ovarian failure (POF) is defined as loss of ovarian function before the age of 40 and affects 1% of women [75]. Etiology is heterogenous and comprises genetic, autoimmune, iatrogenic, and unknown causes. A careful evaluation of the medical history including medical treatments, previous surgery or hereditary disorders is recommended. Laboratory findings are an elevated FSH > 30 mIU/ml, determined twice with 4–6 weeks in between. AMH is of no benefit for the final diagnosis [1, 2].

All types of menstrual disturbances and various climacteric symptoms can be found. Women with POI/POF have a higher risk for estrogen-dependent diseases. However, there is no prospective study which shows lower mortality by hormonal substitution. It might make sense to continue hormonal treatment in women with POF until the average age of menopause.

There seems to be no difference between a hormonal substitution with HRT or contraceptive pills, although two studies reported a small difference in blood pressure in favour of HRT compared to contraceptive pills [76, 77]. Both treatment options can be discussed with POI/POF patients. In women > 40 years, HRT should be preferred.

Women with POI/POF should be informed about the following facts:

- HRT might have a positive effect on blood pressure compared to contraceptive pills.
- HRT and contraceptive pills have a positive effect on bone health.
- HRT does not offer protection from pregnancies.
- Women with POI and contraindications for HRT should be advised about cardiovascular risks, bone health and alternative treatments of climacteric symptoms.
- Women with POI should be generously transferred to specialized centres.

## Informing women about HRT

Women should be informed about risks and benefits of medical interventions. The communication should include the probabilities of expected benefits and possible risks of harm with the patient and possibly with an accompanying person. For an individual assessment and evaluation of the probability of benefit and the risk of harm of HRT individual factors such as the woman´s general state of health, age at menopause, previous HRT, duration and use, doses and type of HRT and diseases while using HRT should be taken into consideration. To give adequate information about the risks to the woman seeking advice, the doctor must be familiar with the principles of risk calculation. He or she should also be able to communicate the probabilities in such a way that the patient can make her own individual decision for or against the initiation of HT. The figures necessary for this communication can be found in the long version and in Table 1 [1].

**Table 1:** Estimated event rate difference associated with EPT or ET vs. placebo in postmenopausal women

Events	Difference of events per 10,000 women years (95% CI)^a^	
	EPT oral	ET oral^b^
Breast cancer	9 (1 bis 19)	− 7 (− 14 bis 0.4)
Coronary heart disease	8 (0 bis 18)	− 3 (− 12 bis 8)^c^
Stroke	9 (2 bis 19)	11 (2 bis 23)^c^
Thromboembolism	21 (12 bis 33)	11 (3 bis 22)^c^
Dementia	22 (4 bis 53)	12 (− 4 bis 41)
Gallbladder disease	21 (10 bis 34)	30 (16 bis 48)
Urinary incontinence	876 (606 bis 1168)	1261 (880 bis 1689)
Bowel cancer	− 6 (− 9 bis − 1)	2 (− 3 bis 10)
Ovarian cancer	2 (− 1 bis 6)	No data
Lung cancer	1 (− 4 bis 7)	1 (− 4 bis 8)
Fractures (osteoporosis)	− 44 (− 71 bis − 13)	− 53 (− 69 bis − 39)
Diabetes	− 14 (− 24 bis − 3)	− 19 (− 34 bis − 3)
Mortality	1 (− 9 bis 12)	1 (− 10 bis 14)

^a^If confidence intervall includes 1, the result is not statistically significant

^b^Estrogen alone only in hysterectomised women

^c^For transdermal ET in doses from up to 50 µg/day observational studies did not demonstrate an influence of the risk for coronary heart disease, stroke or thromboembolism (modified table 3, Gartlehner et al. JAMA 2017)

### Electronic supplementary material

Below is the link to the electronic supplementary material.

Supplementary file1 (DOCX 24 kb)

## Appendix

Detailed information on the development and methodology of the guideline following the criteria of the AWMF can be found on the AWMF homepage (see [1]).

### Guideline group

Coordinating guideline author in charge.

**Table:**

Author	Association of Scientific Medical Societies (AWMF)
Prof. Dr. Olaf Ortmann	Deutsche Gesellschaft für Gynäkologie und Geburtshilfe (DGGG) German Society of Gynecology

### Guideline group

**Table:**

DGGG-Working Group AWMF/Non-AWMF-Scientific Society, Organisation
Arbeitsgemeinschaft Gynäkologische Onkologie (AGO)
Arbeitsgemeinschaft für Urogynäkologie und plastische Beckenbodenrekonstruktion (AGUB)
Berufsverband der Frauenärzte (BVF)
D·A·CH-Gesellschaft Herz-Kreislauf-Prävention
Deutsche Gesellschaft für Allgemeinmedizin und Familienmedizin (DEGAM)
Deutsche Gesellschaft für Angiologie, Gesellschaft für Gefäßmedizin (DGA)
Deutsche Gesellschaft für Endokrinologie (DGE)
Deutsche Gesellschaft für Gynäkologische Endokrinologie und Fortpflanzungsmedizin (DGGEF)
Deutsche Gesellschaft für Gynäkologie und Geburtshilfe (DGGG)
Deutsche Gesellschaft für Hämatologie und Medizinische Onkologie (DGHO)
Deutsche Gesellschaft für Innere Medizin (DGIM)
Deutsche Gesellschaft für Kardiologie – Herz- und Kreislaufforschung (DGK)
Deutsche Gesellschaft für Neurologie (DGN)
Deutsche Gesellschaft für Pharmakologie (DGP)
Deutsche Gesellschaft für psychosomatische Frauenheilkunde und Geburtshilfe (DGPFG)
Deutsche Gesellschaft für Psychiatrie und Psychotherapie, Psychosomatik und Nervenheilkunde (DGPPN)
Deutsche Gesellschaft für Senologie (DGS)
Deutsche Krebsgesellschaft (DKG)
Deutsche Menopause Gesellschaft (DMG)
Dachverband Osteologie (DVO)
European Menopause and Andropause Society (EMAS)
Frauenselbsthilfe nach Krebs
Gesellschaft für Phytotherapie (GPT)
International Menopause Society (IMS)
Österreichische Gesellschaft für Gynäkologie und Geburtshilfe (OEGGG)
Schweizerische Gesellschaft für Gynäkologie und Geburtshilfe (SGGG)

**Table:**

Author Delegate	DGGG-Working Group AWMF/Non-AWMF-Scientific Society, Organisation
Dr. med. C. Albring	BVF, member of the steering committee
Prof. Dr. E. Baum	DEGAM
Dr. med. M. Beckermann	DGPFG
Prof. Dr. K. Bühling	D·A·CH
Prof. Dr. G. Emons	DGGG
Prof. Dr. T. Gudermann	DGP
Prof. Dr. P. Hadji	DVO
Prof. Dr. B. Imthurn	SGGG
PD Dr. med. E.C. Inwald	2nd guideline coordinator
Prof. Dr. L. Kiesel	DMG, member of the steering committee
Prof. Dr. D. Klemperer	Expert, patient information
Dr. P. Klose	GPT
Dr. med. K. König	BVF
Prof. Dr. S. Krüger	DGPPN
Prof. Dr. J. Langhorst	GPT
Prof. Dr. M. Leitzmann	Expert, Epidemiology
Prof. Dr. A. Ludolph	DGN
Prof. Dr. D. Lüftner	DGHO
Frau D. Müller	Frauenselbsthilfe nach Krebs
Prof. Dr. J. Neulen	DGGEF
Dr. med. M. Nothacker	AWMF
Prof. Dr. O. Ortmann	Guideline coordinator, principle author of the guideline, member of the steering committee
Prof. Dr. E. Petri (deceased 21.09.2019)	AGUB
Dr. med. H. Prautzsch	DEGAM
Prof. Dr. F. Regitz-Zagrosek	DGK
Dr. med. K. Schaudig	Expert, Gynecologic Endocrinology
Prof. Dr. F. Schütz	DGS
Dr. med. A. Schwenkhagen	Expert, Gynecologic Endocrinology
Prof. Dr. T. Strowitzki	DGE
Prof. Dr. P. Stute	EMAS, member of the steering committee
Prof. Dr. B.-M. Taute	DGA
Prof. Dr. C. Tempfer	AGO
Prof. Dr. C. von Arnim	DGN
Prof. Dr. L. Wildt	OEGGG
Prof. Dr. E. Windler	DGIM, member of the steering committee

### Statements and recommendations

Grade of recommendation:

A: strong recommendation.
B: recommendation with moderate obligation.
0: open recommendation with low obligation.

Degree of consensus:

+++: strong consensus (> 95% of delegates agree).
++: consensus (> 75 to 95% of delegates agree).
+: majority agrees (> 50 to 75% of delegates agree).
−: no consensus (< 51% of delegates agree).
LLA: Leitlinienadaptation, guideline adaptation.

*Dissenting opinion of the German Society for Phytotherapy (GPT):

The GPT does not support the undifferentiated recommendation on the use of cimicifuga, the level of evidence and grade of recommendation. In contrast to other cimicifuga products (e.g. food supplements), cimicifuga medical products with marketing authorizations have proven their usefulness. Only these should be recommended.

Grade of recommendation:

- A (isopropanolic Cimicifuga medicinal products).
- B (ethanolic Cimicifuga medicinal products)

Level of evidence:

- 1b (isopropanolic Cimicifuga medicinal products).
- 2b (ethanolic Cimicifuga medicinal products)

For the full text on the position of the GPT see electronic supplementary material.

## References

[1] S3 Level, AWMF Registry No. 015-062 (2020) Peri- and postmenopause—diagnosis and interventions. Guideline of the DGGG, SGGG and OEGGG. https://www.awmf.org/leitlinien/detail/ll/015-062.html 10.1055/a-1361-1948 PMC8216766 34168377

[2] National Institute for Health and Care Excellence (NICE) Guideline (2015) Menopause Full Guideline, Methods, evidence and recommendations, Version 1.5. https://www.nice.org.uk/guidance/ng23/evidence/full-guideline-pdf-559549261

[3] Nelson HD, Haney E, Humphrey L, Miller J, Nedrow A, Nicolaidis C, Vesco K, Walker M, Bougatsos C, Nygren P (2005). Management of menopause-related symptoms. Evid Rep Technol Assess.

[4] Avis NE, Crawford SL, Greendale G, Bromberger JT, Everson-Rose SA, Old EB, Hess R, Joffe H, Kravitz HM, Tepper PG, Thurston RC (2015). Duration of menopausal vasomotor symptoms over the menopause transition. JAMA Intern Med..

[5] AWMF Registernummer: 032/034-OL (2018) Diagnostik, Therapie und Nachsorge der Patientinnen mit Endometriumkarzinom, Langversion 1.0. http://www.leitlinienprogramm-onkologie.de/leitlinien/endometriumkarzinom/

[6] Cody JD, Jacobs ML, Richardson K, Moehrer B, Hextall A. Oestrogen therapy for urinary incontinence in post-menopausal women. Cochrane Database Syst Rev 2012 10.1002/14651858.CD001405.pub3 PMC7086391 23076892

[7] AWMF Registernummer: 015/005 (2015) Interdisziplinäre S2e-Leitlinie für die Diagnostik und Therapie der Belastungsinkontinenz der Frau, Langversion 2015. https://www.awmf.org/uploads/tx_szleitlinien/015_005l_S2e_Belastungsinkontinenz_2013-07.pdf

[8] Rahn DD, Carberry C, Sanses TV, Mamik MM (2014). Vaginal estrogen for genitourinary syndrome of menopause—a systematic review. Obstet Gynecol.

[9] Andersson KE, Chapple CR, Cardozo L, Cruz F, Gratzke C, Lee KS, Tannenbaum C, Wein AL (2013) Pharmacological treatment of urinary incontinence. In: Incontinence—5th int consult on incont ICUD-EAU, pp 625–728

[10] Lucas MG, Bosch RJL, Burkhard FC, Cruz F, Madden TB, Nambiar AK, Neisius A, de Ridder DJMK, Tubaro A, Turner WH, Pickard RS (2012). EAU guidelines on assessment and nonsurgical management of urinary incontinence. Eur Urol.

[11] Clinical guideline (2013) Urinary incontinence in women: management. nice.org.uk/guidance/cg171

[12] Lüthje P, Hirschberg AL, Brauner A (2014). Estrogenic action on innate defense mechanisms in the urinary tract. Maturitas.

[13] Wang C, Symington JW, Ma E, Cao B, Mysorekar IU (2013). Estrogenic modulation of uropathogenic *Escherichia coli* in fection pathogenesis in a murine menopause model. Infect Immunity.

[14] AWMF Registernummer: 043/044 (2017) Leitlinienprogramm DGU: Interdisziplinare S3 Leitlinie: Epidemiologie, Diagnostik, Therapie, Pravention und Management unkomplizierter, bakterieller, ambulant erworbener Harnwegsinfektionen bei erwachsenen Patienten. Langversion 1.1–2. http://www.awmf.org/uploads/tx_szleitlinien/043-044l_S3_Harnwegsinfektionen

[15] Beerepoot MA, Geerlings SE, van Haarst EP, van Charante NM, ter Riet G (2013). Nonantibiotic prophylaxis for recurrent urinary tract infections: a systematic review and meta-analysis of randomized controlled studies. J Urol.

[16] Perrotta C, Aznar M, Mejia R, Albert X, Ng CW (2008) Oestrogens for preventing recurrent urinary tract infection in postmenopausal women. Cochrane Database Syst Rev (2):CD005131 10.1002/14651858.CD005131.pub2 PMC13598824 18425910

[17] Manson JE, Hsia J, Johnson KC, Rossouw JE, Assaf AR, Lasser NL, Trevisan M, Black HR, Heckbert SR, Detrano R, Strickland OL, Wong ND, Crouse JR, Stein E, Cushman M, Women's Health Initiative Investigators (2003). Estrogen plus progestin and the risk of coronary heart disease. N Engl J Med.

[18] Harman SM, Black DM, Naftolin F, Brinton EA, Budoff MJ, Cedars MI, Hopkins PN, Lobo RA, Manson JE, Merriam GR, Miller VM, Neal-Perry G, Santoro N, Taylor HS, Vittinghoff E, Yan M, Hodis HN (2014). Arterial imaging outcomes and cardiovascular risk factors in recently menopausal women: a randomized trial. Ann Intern Med.

[19] Canonico M, Oger E, Plu-Bureau G, Conard J, Meyer G, Lévesque H, Trillot N, Barrellier MT, Wahl D, Emmerich J, Scarabin PY, Estrogen and Thromboembolism Risk (ESTHER) Study Group (2007). Hormone therapy and venous thromboembolism among postmenopausal women: impact of the route of estrogen administration and progestogens: the ESTHER study. Circulation.

[20] Canonico M, Fournier A, Carcaillon L, Olié V, Plu-Bureau G, Oger E, Mesrine S, Boutron-Ruault MC, Clavel-Chapelon F, Scarabin PY (2010). Postmenopausal hormone therapy and risk of idiopathic venous thromboembolism: results from the E3N cohort study. Arterioscler Thromb Vasc Biol.

[21] Sweetland S, Beral V, Balkwill A, Liu B, Benson VS, Canonico M, Green J, Reeves GK, Million Women Study Collaborators (2012). Venous thromboembolism risk in relation to use of different types of postmenopausal hormone therapy in a large prospective study. J Thromb Haemost..

[22] Manson JE, Chlebowski RT, Stefanick ML, Aragaki AK, Rossouw JE, Prentice RL, Anderson G, Howard BV, Thomson CA, LaCroix AZ, Wactawski-Wende J, Jackson RD, Limacher M, Margolis KL, Wassertheil-Smoller S, Beresford SA, Cauley JA, Eaton CB, Gass M, Hsia J, Johnson KC, Kooperberg C, Kuller LH, Lewis CE, Liu S, Martin LW, Ockene JK, O'Sullivan MJ, Powell LH, Simon MS, Van Horn L, Vitolins MZ, Wallace RB (2013). Menopausal hormone therapy and health outcomes during the intervention and extended poststopping phases of the Women's Health Initiative randomized trials. JAMA.

[23] Hulley S, Grady D, Bush T, Furberg C, Herrington D, Riggs B, Vittinghoff E (1998). Randomized trial of estrogen plus progestin for secondary prevention of coronary heart disease in postmenopausal women. Heart and Estrogen/progestin Replacement Study (HERS) Research Group. JAMA.

[24] Sare GM, Gray LJ, Bath PM (2008). Association between hormone replacement therapy and subsequent arterial and venous vascular events: a meta-analysis. Eur Heart J.

[25] Boardman HM, Hartley L, Eisinga A, Main C, Roqué i Figuls M, Bonfill Cosp X, Gabriel-Sanchez R, Knight B (2015). Hormone therapy for preventing cardiovascular disease in post-menopausal women. Cochrane Database Syst Rev..

[26] Mohammed K, Abu Dabrh AM, Benkhadra K, Al Nofal A, Carranza Leon BG, Prokop LJ, Montori VM, Faubion SS, Murad MH (2015). Oral vs transdermal estrogen therapy and vascular events: a systematic review and meta-analysis. J Clin Endocrinol Metab.

[27] Bath PM, Gray LJ (2005). Association between hormone replacement therapy and subsequent stroke: a meta-analysis. BMJ.

[28] Gu H, Zhao X, Zhao X, Yang Y, Lv X (2014). Risk of stroke in healthy postmenopausalwomen during and after hormone therapy: a meta-analysis. Menopause.

[29] Wells G, Tugwell P, Shea B, Guyatt G, Peterson J, Zytaruk N (2002). Meta-analyses of therapies for postmenopausal osteoporosis. V. Meta-analysis of the efficacy of hormone replacement therapy in treating and preventing osteoporosis in postmenopausal women. Endocr Rev.

[30] Torgerson DJ, Bell-Syer SE (2001). Hormone replacement therapy and prevention of nonvertebral fractures: a meta-analysis of randomized trials. JAMA.

[31] Yu X, Zhou S, Wang J, Zhang Q, Hou J, Zhu L, He Y, Zhao J, Zhong S (2017). Hormone replacement therapy and breast cancer survival: a systematic review and meta-analysis of observational studies. Breast Cancer.

[32] AWMF-Register-Nr.: 183/001 (2017) Prophylaxe, Diagnostik und Therapie der OSTEOPOROSE bei postmenopausalen Frauen und Männern. Leitlinie des Dachverbands der Deutschsprachigen Wissenschaftlichen Osteologischen Gesellschaften e.V.

[33] (2015) DGPPN, BÄK, KBV, AWMF (Hrsg.) für die Leitliniengruppe Unipolare Depression*. S3-Leitlinie/Nationale VersorgungsLeitlinie Unipolare Depression—Langfassung, 2. Auflage. Version 5. 10.6101/AZQ/000364. www.depression.versorgungsleitlinien.de

[34] Colvin A, Richardson GA, Cyranowski JM, Youk A, Bromberger JT (2017). The role of family history of depression and the menopausal transition in the development of major depression in midlife women: study of women's health across the nation mental health study (SWAN MHS). Depress Anxiety.

[35] Prentice RL, Manson JE, Langer RD, Anderson GL, Pettinger M, Jackson RD, Johnson KC, Kuller LH, Lane DS, Wactawski-Wende J, Brzyski R, Allison M, Ockene J, Sarto G, Rossouw JE (2009). Benefits and risks of postmenopausal hormone therapy when it is initiated soon after menopause. Am J Epidemiol.

[36] Cordina-Duverger E, Truong T, Anger A, Sanchez M, Arveux P, Kerbrat P, Guénel P (2013). Risk of breast cancer by type of menopausal hormone therapy: a case-control study among post-menopausal women in France. PLoS ONE.

[37] Manson JE, Aragaki AK, Rossouw JE (2017). Menopausal hormone therapy and long-term all-cause and cause-specific mortality the Women’s Health Initiative randomized trials. JAMA.

[38] Anderson GL, Limacher M, Assaf AR, Bassford T, Beresford SA, Black H, Bonds D, Brunner RL, Brzyski RG, Caan BJ (2004). Effects of conjugated equine estrogen in postmenopausal women with a hysterectomy. JAMA.

[39] Hodis HN, Mack WJ, Lobo RA, Shoupe D, Sevanian A, Mahrer PR, Selzer RH, Liu CR, Liu CH, Azen SP (2001). Estrogen in the prevention of atherosclerosis: a randomized, double-blind, placebo-controlled trial. Ann Intern Med.

[40] Viscoli CM, Brass LM, Kernan WN, Sarrel PM, Suissa S, Horwitz RI (2001). A clinical trial of estrogen-replacement therapy after ischemic stroke. N Engl J Med.

[41] Cherry N, Gilmour K, Hannaford P, Heagarty A, Khan MA, Kitchener HC, McNamee R, Elstein M, Kay C, Seif M (2002). Oestrogen therapy for prevention of reinfarction in postmenopausal women: a randomised placebo controlled trial. Lancet.

[42] Moegele M, Buchholz S, Seitz S, Ortmann O (2012). Vaginal estrogen therapy in postmenopausal breast cancer patients treated with aromatase inhibitors. Arch Gynecol Obstet..

[43] Holmberg L, Anderson H (2004). HABITS (hormonal replacement therapy after breast cancer—is it safe?), a randomised comparison: trial stopped. Lancet.

[44] Holmberg L, Iversen OE, Rudenstam CM, Hammar M, Kumpulainen E, Jaskiewicz J, Jassem J, Dobaczewska D, Fjosne HE, Peralta O, Arriagada R, Holmqvist M, Maenpaa J, HABITS Study Group (2008). Increased risk of recurrence after hormone replacement therapy in breast cancer survivors. J Natl Cancer Inst.

[45] Farquhar CM, Marjoribanks J, Lethaby A, Lamberts Q, Suckling JA, the Cochrane HT Study Group (2005) Long-term hormone therapy for perimenopausal and postmenopausal women. Cochrane Database Syst Rev (3):CD004143 **(Review. Update in: Cochrane Database Syst Rev. 2009;(2):CD004143)** 10.1002/14651858.CD004143.pub2 16034922

[46] Collins JA, Blake JM, Crosignani PG (2005). Breast cancer risk with postmenopausal hormonal treatment. Hum Reprod Update.

[47] Kenemans P, Bundred NJ, Foidart JM, Kubista E, von Schoultz B, Sismondi P, Vassilopoulou-Sellin R, Yip CH, Egberts J, Mol-Arts M, Mulder R, van Os S, Beckmann MW (2009). Safety and efficacy of tibolone in breast-cancer patients with vasomotor symptoms: a double-blind, randomised, non-inferiority trial. Lancet Oncol.

[48] Donders G (2014). Ultra-low-dose estriol and Lactobacillus acidophilus vaginal tablets (Gynoflor®) for vaginal atrophy in postmenopausal breast cancer patients on aromatase inhibitors: pharmacokinetic, safety, and efficacy phase I clinical study. Breast Cancer Res Treat.

[49] Grady D, Gebretsadik T, Kerlikowske K, Ernster V, Petitti D (1995). Hormone replacement therapy and endometrial cancer risk: a meta-analysis. Obstet Gynecol.

[50] Nelson HD, Humphrey LL, Nygren P, Teutsch SM, Allan JD (2002). Postmenopausal hormone replacement therapy: scientific review. JAMA.

[51] Lacey JV, Brinton LA, Lubin JH, Sherman ME, Schatzkin A, Schairer C (2005). Endometrial carcinoma risks among menopausal estrogen plus progestin and unopposed estrogen users in a cohort of postmenopausal women. Cancer Epidemiol Biomark Prev.

[52] Allen NE, Tsilidis KK, Key TJ, Dossus L, Kaaks R, Lund E, Bakken K, Gavrilyuk O, Overvad K, Tjonneland A, Olsen A, Fournier A, Fabre A, Clavel-Chapelon F, Chabbert- Buffet N, Sacerdote C, Krogh V, Bendinelli B, Tumino R, Panico S, Bergmann M, Schuetze M, van Duijnhoven FJB, Bueno-de-Mesquita H, Onland-Moret NC, van Gils CH, Amiano P, Barricarte A, Chirlaque MD, Molina-Montes ME, Redondo ML, Duell EJ, Khaw KT, Wareham N, Rinaldi S, Fedirko V, Mouw T, Michaud DS, Riboli E (2010). Menopausal hormone therapy and risk of endometrial carcinoma among postmenopausal women in the European Prospective Investigation into cancer and nutrition. Am J Epidemiol.

[53] Beral V, Bull D, Reeves G, Million Women Study Collaborators (2005). Endometrial cancer and hormone-replacement therapy in the Million Women Study. Lancet.

[54] Razavi P, Pike MC, Horn-Ross PL, Templeman C, Bernstein L, Ursin G (2010). Long-term postmenopausal hormone therapy and endometrial cancer. Cancer Epidemiol Biomark Prev.

[55] Fournier A, Dossus L, Mesrine S, Vilier A, Boutron-Ruault MC, Clavel-Chapelon F, Chabbert-Buffet N (2014). Risks of endometrial cancer associated with different hormone replacement therapies in the E3N cohort, 1992–2008. Am J Epidemiol.

[56] Chlebowski R (2015). Aromatase inhibitors, tamoxifen, and endometrial cancer in breast cancer survivors. Cancer.

[57] Shim SH, Lee SJ, Kim SN (2014). Effects of hormone replacement therapy on the rate of recurrence in endometrial cancer survivors: a meta-analysis. Eur J Cancer.

[58] Manley K, Edey K, Braybrooke J, Murdoch J (2012). Hormone replacement therapy after endometrial cancer. Menopause Int.

[59] Felix AS, Arem H, Trabert B, Gierach GL, Park Y, Pfeiffer RM, Brinton LA (2015). Menopausal hormone therapy and mortality among endometrial cancer patients in the NIH-AART Diet and Health Study. Cancer Causes Control.

[60] Lester J, Pahouja G, Andersen B, Lustberg M (2015). Atrophic vaginitis in breast cancer survivors: a difficult survivorship issue. J Pers Med.

[61] Lee YK, Chung HH, Kim JW, Park NH, Song YS, Kang SB (2011). Vaginal pH-balanced gel for the control of atrophic vaginitis among breast cancer survivors: a randomized con- trolled trial. Obstet Gynecol.

[62] Pitkin RM, VanVoorhis LW (1971). Postirradiation vaginitis. An evaluation of prophylaxis with topical estrogen. Radiology.

[63] Singh P, Oehler MK (2010). Hormone replacement after gynaecological cancer. Maturitas.

[64] Collaborative Group on Epidemiological Studies of Ovarian Cancer. Menopausal hormone use and ovarian cancer risk: individual participant meta-analysis of 52 epidemiological studies. Lancet 385:1835–42 10.1016/S0140-6736(14)61687-1 PMC4427760 25684585

[65] Guidozzi F, Daponte A (1999). Estrogen replacement therapy for ovarian carcinoma survivors: a randomized controlled trial. Cancer.

[66] Eeles RA (1991). Hormone replacement therapy and survival after surgery for ovarian cancer. BMJ.

[67] Eeles RA (2015). Adjuvant hormone therapy may improve survival in epithelial ovarian cancer: results of the AHT randomized trial. J Clin Oncol.

[68] Ursic-Vrscaj M, Bebar S, Zakelj MP (2001). Hormone replacement therapy after invasive ovarian serous cystadenocarcinoma treatment: the effect on survival. Menopause.

[69] Mascarenhas C (2006). Use of hormone replacement therapy before and after ovarian cancer diagnosis and ovarian cancer survival. Int J Cancer.

[70] AWMF-Registernummer: 032/035OL (2016). S3-Leitlinie Diagnostik, Therapie und Nachsorge maligner Ovarialtumoren, Langversion 2.0. https://leitlinienprogramm-onkologie.de/Ovarialkarzinom.61.0.html

[71] Li D, Ding CY, Qiu LH (2015). Postoperative hormone replacement therapy for epithelial ovarian cancer patients: a systematic review and meta-analysis. Gynecol Oncol.

[72] Lin KJ, Cheung WY, Lai JY (2012). The effect of estrogen vs. combined estrogen-progestin therapy on the risk of colorectal cancer. Int J Cancer.

[73] Mørch LS, Lidegaard Ø, Keiding N, Løkkegaard E, Kjær SK (2016). The influence of hormone therapies on colon and rectal cancer. Eur J Epidemiol.

[74] Chan JA, Meyerhardt JA, Chan AT, Giovannucci EL, Colditz GA, Fuchs CS (2006). Hormone replacement therapy and survival after colorectal cancer diagnosis. J Clin Oncol.

[75] Coulam CB, Adamson SC, Annegers JF (1986). Incidence of premature ovarian failure. Obstet Gynecol.

[76] Guttmann H, Weiner Z, Nikolski E, Ish-Shalom S, Itskovitz-Eldor J, Aviram M, Reisner S, Hochberg Z (2001). Choosing an oestrogen replacement therapy in young adult women with Turner syndrome. Clin Endocrinol.

[77] Langrish JP, Mills NL, Bath LE, Warner P, Webb DJ, Kelnar CJ, Critchley HO, Newby DE, Wallace WH (2009). Cardiovascular effects of physiological and standard sex steroid replacement regimens in premature ovarian failure. Hypertension.

