# Addendum to: Peri- and postmenopause—diagnosis and interventions interdisciplinary S3 guideline of the association of the scientific medical societies in Germany (AWMF 015/062): short version

**DOI:** 10.1007/s00404-021-06009-7

**Published:** 2021-03-16

**Authors:** Olaf Ortmann, Maria J. Beckermann, Elisabeth C. Inwald, Thomas Strowitzki, Eberhard Windler, Clemens Tempfer, for the Guideline Group

**Affiliations:** 1 Department of Gynecology and Obstetrics, University Medical Center Regensburg, Landshuter Straße 65, 93053 Regensburg, Germany; 2 Frauenärztliche Gemeinschaftspraxis, Köln, Germany; 3 Department of Gynecological Endocrinology and Fertility Disorders, University Women’s Hospital, Heidelberg, Germany; 4 Endocrinology and Metabolism of Ageing, University Medical Center Hamburg-Eppendorf, Hamburg-Eppendorf, Germany; 5 Department of Obstetrics and Gynecology, Ruhr-University Bochum, Bochum, Germany

## Addendum to: Archives of Gynecology and Obstetrics 10.1007/s00404-020-05682-4

The interdisciplinary S3 guideline, Peri- and postmenopause—diagnosis and interventions “of the association of the scientific medical societies in Germany (AWMF 015/062) was published in January 2020 and a short version in July 2020 (O. Ortmann et al. Arch Gynecol Obstet 302:763–777).

This guideline did not include the meta-analysis performed by the Collaborative Group on Hormonal Factors in Breast Cancer of data from prospective and retrospective observational and randomized studies on the association between peri- and postmenopausal hormone therapy (HT) and breast cancer risk. Since evidence from the meta-analysis of the Collaborative Group on Hormonal Factors in Breast Cancer is relevant, authors of the S3 guideline Peri- and postmenopause – diagnosis and interventions “ wrote an addendum on behalf of the steering committee that evaluates the data [1].

The authors of the S3 guideline propose that numbers quoted in Table 1 are appropriate for counseling women with climacteric symptoms. Five years of a sequential combined HT containing estrogen and progestin (EPT) started from the age of 50 years lead to 14 additional breast cancer cases per 1.000 women within the next 20 years. A continuously combined EPT causes 20 additional breast cancer cases whereas a HT containing only estrogen (ET) leads to 5 additional cases. These risk estimates are consistent with data published previously. Changes of statements or recommendations given in the S3 guideline, Peri- and postmenopause—diagnosis and interventions are, therefore, not required.

**Table 1:** Breast cancer risk associated with HT

HT	Additional breast cancer cases per 1.000 women at ages ≥ 50 years after 5 years of HT within 20 years	Additional breast cancer cases per 1.000 women at ages ≥ 50 years after 10 years of HT within 20 years
Sequential EPT	+ 14	+ 29
Continuously-combined EPT	+ 20	+ 40
ET	+ 5	+ 11

Based on data from the meta-analysis by the Collaborative Group on Hormonal Factors in Breast Cancer

*HT* hormone therapy, *EPT* estrogen-progestin therapy, *ET* estrogen therapy

Table 2 includes numbers that are suitable to counsel women regarding the influence of the duration of a HT on breast cancer risk. Results from the meta-analysis show that an ET for up to 4 years does not increase breast cancer risk within the following 9 years (relative risk [RR] 1.07; 95% confidence interval [CI] 0.96–1.20). Also, sequential or continuously combined EPT for up to 4 years do not increase breast cancer risk within the following 9 years (RR 1.06; 95% CI 0.98–1.15) (Table 2). However, data from the meta-analysis indicate an increased breast cancer risk after 1 year of ET or EPT (Table 2). It is unclear whether this results from HT use or detection bias.

**Table 2:** Breast cancer risk by duration of HT

HT	Relative breast cancer risk during HT	Relative breast cancer risk up to 9 years after cessation of HT
ET for 1–4 years	RR 1.17; 95% CI 1.10–1.26	RR 1.07; 95% CI 0.96–1.20
EPT (continuously-combined or sequential) for 1–4 years	RR 1.60; 95% CI 1.52–1.69	RR 1.06; 95% CI 0.98–1.15

Based on data from the meta-analysis by the Collaborative Group on Hormonal Factors in Breast Cancer

Degree of consensus for the addendum: strong consensus

*HT* hormone therapy, *EPT* estrogen-progestin therapy, *ET* estrogen therapy, *RR* relative risk, *CI* confidence interval
