# Supplementary material for: Peri- and postmenopause—diagnosis and interventions interdisciplinary S3 guideline of the association of the scientific medical societies in Germany (AWMF 015/062): short version
Source: Arch Gynecol Obstet. 2020 Jul 13;302(3):763–77. doi: 10.1007/s00404-020-05682-4 (PMC7447675; doi:10.1007/s00404-020-05682-4)
Supplement: Supplementary file 1 — Supplementary file1 (DOCX 24 kb) [file 404_2020_5682_MOESM1_ESM.docx]

**Position of the German Society for Phytotherapy
(*Gesellschaft für Phytotherapie e.V.)* (GPT)**

July 18, 2019

This position (dissenting opinion) regards the S3 Guideline 015-062, Chapter 1.4, therein the table of recommendations, the table 10 of benefits and risks, and the corresponding text on Cimicifuga racemosa.

The GPT does not support the undifferentiated recommendations and corresponding statements with regard to level of evidence and grade of recommendation as well as proof of benefit of Cimicifuga. Therefore, the following ‘**Dissenting Opinion** of the Society for Phytotherapy’ is to be integrated in Chapter 1.4, after the table of recommendations and immediately after table 10 of benefits and risks in this Guideline:

In contrast to other Cimicifuga products (e.g. food supplements), Cimicifuga medicinal products with marketing authorizations have proven their usefulness. Therefore, only Cimicifuga medicinal products with marketing authorizations should be recommended.

Grade of Recommendation: A (isopropanolic Cimicifuga medicinal products)

B (ethanolic Cimicifuga medicinal products)

Level of Evidence: 1b (isopropanolic Cimicifuga medicinal products)

2b (ethanolic Cimicifuga medicinal products)

The full justification of this statement can be found in the GPT’s expert opinion from November 26, 2018, on the consultation version as well as the GPT’s expert opinion from June 13, 2019, on the version voted on (reconciliation version). The key points are given below:

**Justification:**

A systematic review found that only Cimicifuga medicinal products, but not other Cimicifuga products, have been shown to be effective in randomized controlled trials (RCTs) [1]: For an HMPC monograph-conform, isopropanolic Cimicifuga medicinal product, 4 RCTs, 2 of which had confirmatory evidence, consistently confirmed its efficacy (LoE 1b, GR A). For each of the three HMPC monograph-conform, ethanolic Cimicifuga medicinal products, there was 1 RCT available, which exploratively demonstrated their efficacy (LoE 2b, GR B). The efficacy of two Cimicifuga-St. John’s wort medicinal product combinations with marketing authorizations was confirmed in 1 RCT each (LoE 1b, GR A). In contrast, the Cimicifuga products without marketing authorizations demonstrated no significant difference compared to placebo treatment in two RCTs. An update of the review with newer clinical studies reconfirmed these results [2]: Once again, all RCTs with medicinal products with marketing authorizations had a positive outcome while the only Cimicifuga product without a marketing authorization did not provide any proof of efficacy.

An undifferentiated recommendation for all Cimicifuga preparations could lead to products being taken where the effectiveness, safety, and pharmaceutical quality have not been proven. This is particularly important with regard to the potential risks of contamination and adulteration found in food supplements [3]. In addition, individual cases of adverse liver events connected to Cimicifuga foods supplements, which have not been tested and, in part, are overdosed, have been reported [4]. Hence, the GPT considers it essential to inform therapists and patients that only Cimicifuga medicinal products, which have been tested in the course of a marketing authorization, but not other Cimicifuga products, should be used to treat vasomotor symptoms.

1. Beer A-M, Neff A: **Differentiated evaluation of extract-specific evidence on Cimicifuga racemosa's efficacy and safety for climacteric complaints**. *Evid Based Complement Alternat Med* 2013:Article ID 860602.

2. Beer A-M: **Cimicifuga racemosa bei klimakterischen Beschwerden - Aktuelle Daten bestätigen Wirksamkeit und Sicherheit**. *Zeitschrift für Phytotherapie* 2015, **36**(01):10-17.

3. Jiang B, Kronenberg F, Nuntanakorn P, Qiu MH, Kennelly EJ: **Evaluation of the botanical authenticity and phytochemical profile of black cohosh products by high-performance liquid chromatography with selected ion monitoring liquid chromatography-mass spectrometry**. *J Agric Food Chem* 2006, **54**(9):3242-3253.

4. Teschke R, Schwarzenboeck A, Schmidt-Taenzer W, Wolff A, Hennermann KH: **Herb induced liver injury presumably caused by black cohosh: a survey of initially purported cases and herbal quality specifications**. *Ann Hepatol* 2011, **10**(3):249-259.
